# Comparative Effects of Various Plasticizers on the Physicochemical Characteristics of Polyhydroxybutyrate (PHB) Film for Food Packaging

**DOI:** 10.3390/polym17223071

**Published:** 2025-11-20

**Authors:** Siwar Taamallah, Sabrine Douiri, Sherif M. A. S. Keshk, Rim Ben Arfi, Achraf Ghorbal, Khaled Charradi, Rached Ben Hassen, Hamadi Attia, Dorra Ghorbel

**Affiliations:** 1 INSAT, University of Carthage, Centre Urbain Nord, B.P. 676, Tunis 1080, Tunisia; siwar.taamallah@insat.ucar.tn (S.T.); sabrine.douiri@enis.tn (S.D.); 2 Food Analysis, Valorization, and Safety Laboratory, LAVASA (LR11ES45), ENIS, University of Sfax, Sfax 3038, Tunisia; hamadi.attia@enis.tn; 3 Become: Technology, Science, AI & Automation Lab., 63 rue de Tolbiac, 75013 Paris, France; sherif.keshk@become.institute; 4 Research Unit Advanced Materials, Applied Mechanics, Innovative Processes, and Environment, 2MPE (UR22ES04), ISSAT Gabes, University of Gabes, Avenue Omar Ibn El Khattab, Gabes 6072, Tunisia; rim.benarfi@issatgb.u-gabes.tn (R.B.A.); achraf.ghorbal@issatgb.u-gabes.tn (A.G.); 5 Nanomaterials and Systems for Renewable Energy Laboratory, Research and Technology Center of Energy, Technopark Borj Cedria, Hammam-Lif 2084, Tunisia; charradikaled@gmail.com; 6 Laboratory of Materials and Environment for Sustainable Development, LR18ES10, ISSBAT, University of Tunis El Manar, 9 Street Zouheir Essafi, Tunis 1006, Tunisia; rached.benhassen@issbat.utm.tn

**Keywords:** PHB, plasticizer, potassium phosphate, soy lecithin, food packaging

## Abstract

This work examined the effects of four plasticizers, glycerol (GLY), potassium phosphate (PHOS), polyethylene glycol (PEG), and soy lecithin (SL), on the structural, surface, thermal, optical, and mechanical properties of polyhydroxybutyrate (PHB) films. FTIR spectra demonstrated that these plasticizers maintained the PHB molecular structure, while X-ray diffraction data proved that PHB crystallinity decreased upon adding SL, GLY, and PHOS. Under SEM, we discovered several defects in the plasticized samples, most of which were holes of distinct sizes and forms. The thermal analyses evaluated the impact of plasticization on PHB thermal processability, demonstrating that the material’s thermal stability improved, easing thermal processing due to the reduced melting peak temperatures (T_m_) caused by all the additives assessed. While PEG, GLY, and PHOS reduced the hydrophilicity of the film, SL enhanced its affinity to water, as shown by the contact angle measurements. Reduced transparency resulted from adding 20% plasticizers with an increase of 345% in elongation at break and a decrease of 67% in elastic modulus compared to pristine PHB. Thus, SL proved to be the most promising of the four plasticizers used in terms of mechanical properties, crucial for PHB-based films for food packaging.

## 1. Introduction

The high molecular weight and hydrophobic nature of petroleum-based synthetic polymers have led to their widespread use in various applications. However, environmental concerns surrounding their pervasive utilization have sparked a growing interest in biodegradable alternatives derived from renewable resources. Indeed, extensive efforts have been targeted toward developing biodegradable plastics, with environmental conservation as the primary focus [1]. In the production of biodegradable plastics, incorporating additives into the polymeric matrix is critical for optimizing the final properties and producing materials with diverse practicalities and valuable attributes [2]. These additives, classified according to their functions rather than chemical structure, encompass plasticizers [3], reinforcing fillers, and impact modifiers that alter the mechanical characteristics of biopolymers and loads [4], diluents, and extenders that help reduce the cost [5]. Surface characteristics can be modified using anti-static compounds, anti-slip additives, anti-wear additives, and adhesion boosters [6]. In contrast, optical characteristics are altered typically with pigments, dyes, and nucleating agents [7]. Stabilizers regulate polymer aging, and foaming agents and flame retardants offer further enhancements [8]. Polyhydroxyalkanoates (PHAs) are a class of biopolymers synthesized by microorganisms as a survival mechanism under specific growth conditions. They pile inside cells as a carbon and energy reserve [9,10]. PHAs, with poly(3-hydroxybutyrate) (PHB) as the most well-characterized polymer, have garnered significant attention due to their commercial potential as biodegradable thermoplastics that could replace conventional petroleum-based polymers. Yet, besides rigidity and brittleness, poly(3-hydroxybutyrate) faces challenges such as a narrow processing temperature window, low toughness, low elongation-at-break, and high production costs [11]. While brittleness may be addressed by lowering the processing temperature or adopting methods such as crosslinking to reform polymer linkages [12], the other deficiencies require the incorporation of plasticizers for efficient solutions. There are various strategies for enhancing PHB properties, with the addition of plasticizers being the predominant approach. Plasticizers are added to synthetic and bio-based polymeric materials to increase their flexibility and toughness while reducing the glass transition temperature. Notable plasticizers for PHB include Lapol 108 (a polyester plasticizer) [13], triacetin, acetyl tributyl citrate [14], poly (ethylene glycol) (PEG) [15], glycerol [16], and limonene [17]. Glycerol and PEG are particularly favored due to their solubility in water and organic solvents, lack of toxicity, and resistance to degradation [18]. While PEG enhances the elongation at break, softness, and mechanical properties of PHB, it reduces its crystallinity [19].

Other plasticizers, such as glycerol (GLY), polyglycerols (PGs), glycerol triacetate (GTA), and glycerol tributyrate (GTB), have also demonstrated effectiveness in improving PHB thermal processability [20]. GTA and GTB exhibited superior mechanical performance, demonstrating enhanced deformation at break and lower maximum tensile strength. In contrast, GLY and PG induced reduced mechanical qualities, impacting the performance of the PHB film; GLY and GTA decreased the barrier feature, while GTB and PGs improved water vapor permeability [20]. However, the impact of soy lecithin (SL) and potassium phosphate (PHOS) on PHB has remained unexplored, specifically in terms of their thermal and mechanical properties and whether their effects are similar to those of PEG or GLY. Hence, the main objective of this study is to evaluate the effectiveness of soy lecithin and potassium phosphate as PHB plasticizers. Soy lecithin is a byproduct of soybean oil production that can be sustainable if sourced from responsibly farmed soybeans (i.e., non-GMO, organic, deforestation-free, etc.). SL is a natural mixture of phospholipids, readily biodegradable in both aerobic and anaerobic environments. However, potassium phosphate is mainly made from mined phosphate rock and has limited sustainability unless sourced from recycled phosphorus. PHOS is an inorganic salt, so it is not considered biodegradable, but it dissolves in water and becomes part of natural phosphorus and potassium cycles; since it can be assimilated by plants, it is therefore bioavailable.

This investigation assessed the effects of SL and PHOS on PHB film morphology, crystalline structure, and its mechanical, optical, and thermal characteristics. The results were compared with those made using PEG and GLY providing a thorough overview.

## 2. Materials and Methods

### 2.1. Raw Materials and Chemicals

Polyhydroxybutyrate (PHB) with a molecular weight of 550,000 g·mol^−1^ was sourced from Goodfellow Cambridge Limited (Huntingdon, UK). Chloroform (CHCl_3_, 99%, M_W_: 119.38 g·mol^−1^) was acquired from AnalaR NORMAPUR. Glycerol (H(OCH_2_CH_2_)_n_ O_4_, M_W_: 92.09 g·mol^−1^) and monobasic potassium phosphate (KH_2_PO_4_, M_W_: 136.09 g·mol^−1^) were supplied by Lobachemie. Soy lecithin (L-α-phosphatidylcholine) was obtained from Sigma-Aldrich. Polyethylene glycol (PEG) with a molecular weight of 4000 g·mol^−1^ was procured from Mi Media Laboratories (Coimbatore, India).

### 2.2. Fabrication of PHB Films

The PHB films were fabricated using a conventional casting/evaporation technique. In this procedure, PHB (2 g) was solubilized in 50 mL of chloroform (99%, *w*/*v*). Solubilization occurred in a water bath at 60 °C for 3 h. Before dissolving PHB in chloroform, plasticizers, including glycerol (GLY), soy lecithin (SL), polyethylene glycol (PEG), and monobasic potassium phosphate (PHOS), were added to the PHB at a concentration of 20 wt.% (based on PHB dry weight). Subsequently, 15 mL of the solution was poured into a 94 mm glass Petri dish and allowed to dry in the captair fume hood for 2 h.

### 2.3. FTIR Spectroscopy

The preliminary structural characterization of the films was conducted using Fourier Transform Infrared (FTIR) spectrometry. The FTIR spectra of the various films were acquired by directly depositing film samples onto the diamond crystal of the Spectrum Two spectrometer (PerkinElmer, Springfield, IL, USA). Each spectrum represents the accumulation of 16 scans. The spectra were acquired in the range of 4000 to 450 cm^−1^ to facilitate a detailed analysis of molecular vibrations at a resolution of 2 cm^−1^ [21].

### 2.4. X-Ray Diffractometry (XRD)

X-ray diffraction analysis was conducted using the RIGAKU PRINT 2200 V series X-ray diffractometer following established procedures [22]. The analysis utilized Ni-filtered CuKα radiation (1.54 Å) at ambient temperature, with operational parameters set at 40 kV and 30 mA.

The crystallinity index (CI) was calculated using Origin Pro 2018 software (version 9.5), as depicted in Equation (1).

(1) $$\mathrm{CI}=\frac{Ac}{Ac+Aa}\times 100$$

Here, Ac represents the sum of the areas under the crystalline peaks extracted from the XRD diffractogram, and Aa denotes the region of the amorphous halo [23].

### 2.5. Scanning Electron Microscopy (SEM)

The microstructural properties of the films were examined using a Thermo Scientific Quanta 250 SEM (Thermo Fisher Scientific, Hillsboro, OR, USA), coupled with the energy-dispersive spectroscopy technique, employing Pathfinder software (version 1.3). To ensure sample stability during testing, specimens were affixed to stubs using adhesive carbon tabs. SEM images were captured with an accelerating voltage set to 20.0 kV [22].

### 2.6. Differential Scanning Calorimetry (DSC)

The thermal properties of PHB films were investigated using a Pyris 6 DSC (Perkin Elmer, Springfield, IL, USA) differential scanning calorimeter. Film samples, weighing 5–10 mg, were placed in aluminum pans and subjected to a heating cycle from room temperature to 200 °C (isothermal for 3 min), followed by cooling to −50 °C (isothermal for 3 min) and reheating to 210 °C. Each cycle involved a 3 min isothermal phase. The analysis was conducted at 20 °C·min^−1^ in an inert nitrogen atmosphere.

The crystallinity index (X_DSC_) was determined using Equation (2) [14,24].

(2) $$X_{\mathrm{DSC}}\;(\%)=\frac{∆H_{m}}{∆H_{m}^{{}^{\circ}}\times w_{i}}\times 100$$

where ∆H_m_ is the melting enthalpy in the sample, $∆H_{m}^{{}^{\circ}}$ is the melting heat for pure crystalline PHB ($∆H_{m}^{{}^{\circ}}$ = 146 J·g^−1^), and w_i_ is the PHB weight fraction in the blend.

### 2.7. Thermogravimetric Analysis (TGA)

Film thermal stability was examined utilizing thermogravimetric analysis (TGA/DSC 3+ Mettler Toledo, Greifensee, Switzerland) under nitrogen, with temperatures ranging from 30 to 800 °C and a heating rate of 10 °C·min^−1^. Origin Pro 2018 software was employed to generate differential thermogravimetry curves through the first derivatives (DTG) of the weight loss rate [25,26].

### 2.8. Contact Angle

A Drop Shape Analyzer DSA25E goniometer (Krüss, Bloomfield, CT, USA), featuring a high-resolution camera and Krüss Advance software, was utilized to measure the water contact angle at 24 °C. Contact angles were determined by randomly depositing ten drops (4 µL) of distilled water onto the film surface, and the average values of ten measurements for each drop were calculated [27].

### 2.9. UV-Vis Spectrophotometry

The transmittance of the PHB films was recorded at 600 nm using a CECIL CE3055 UV-vis Spectrophotometer (Cecil Instruments Ltd., Cambridge, UK) at ordinary temperature. The transparency of the PHB films was determined according to Equation (3) [28].

(3) $$\mathrm{Transparency}=\frac{-log\%T}{d}$$

where %T is the transmittance percentage at 600 nm, and d is the thickness of the film in millimeters.

### 2.10. Mechanical Characterization

The mechanical properties of the films, including tensile strength, elongation at break, and Young’s modulus, were determined using a Universal Testing Machine with a 100 N force sensor (Lloyd Instruments Ltd., Plus/Easy Test, Bognor Regis, UK) coupled to an A/MTG system. Film specimens were cut into bands measuring 2 cm × 7 cm, and their thickness was determined by averaging the measurements taken at ten random points using a FERVI electronic digital micrometer. The films were clamped between two molds with an initial distance of 6 cm. Force data were recorded using Nexigen Plus software (version 4.1.5), and the extension speed was set at 10 mm·min^−1^. Tensile strength (TS, Pa), elongation at break (EAB, %), and Young’s modulus (E, Pa) were calculated using Equations (4)–(7) [22].

(4) $$\mathrm{TS}\;(\mathrm{Pa})=\frac{Maximum\;force}{Film\;section}$$

(5) Film section = width × thickness

(6) $$\mathrm{EAB}\;(\%)=\frac{L-L_{0}}{L_{0}}\times 100$$

(7) $$E=\frac{Curve\;slope}{Film\;section}\;L_{0}$$

Here, L_0_ and L represent the film’s initial and final lengths, respectively. Before mechanical characterization, all samples underwent a 48 h conditioning period at 25 °C and 54% RH.

### 2.11. Statistical Analyses

Statistical analyses were conducted with three replicates for the mechanical tests and ten replicates for the contact angle measurements. The results were presented as mean values (±standard deviation) and analyzed using IBM SPSS software (version 30) through analysis of variance (ANOVA) with a 95% confidence interval. Duncan’s post hoc test assessed the significant differences between the means of the tested factors, where *p* < 0.05 indicated statistical significance.

## 3. Results and Discussion

### 3.1. Structural Characterization

**Figure 1:**
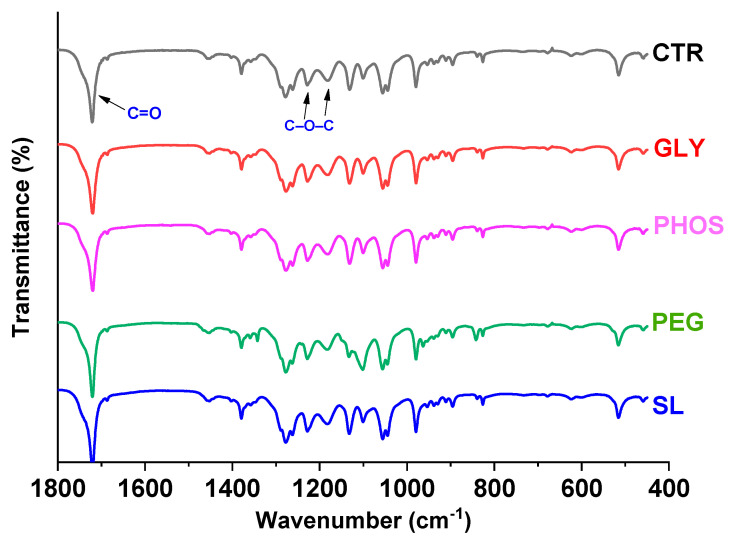
FTIR analysis of PHB films plasticized with 20 wt.% content.

Figure 1 illustrates the ATR-FTIR analysis, demonstrating the effects of four distinguishable plasticizers on polyhydroxybutyrate (PHB). The spectra revealed the characteristic peaks associated with PHB amorphous and crystalline phases. Notably, the bands at 3010 cm^−1^, 2997 cm^−1^, 2975 cm^−1^, 2933 cm^−1^, 2873 cm^−1^, and 1262 cm^−1^ illustrated the crystalline phase, while the peak at 1182 cm^−1^ marked the amorphous phase [29]. Each spectrum exhibited a prominent peak associated with the stretching vibration of the free (amorphous component) and intra- (crystalline component) carbonyl groups (C = O). This characteristic peak appeared as a shoulder around 1740 cm^−1^, indicative of the amorphous phase, and a pronounced peak at 1720 cm^−1^, representing the crystalline phase [30]. The PHB spectrum did not display any characteristic bands associated with hydroxyl (3300 cm^−1^) and amide II (1542 cm^−1^) groups, confirming the effective removal of polypeptide and hydroxyl-containing compounds during the purification process [31]. In the sample containing PEG as the plasticizer, characteristic and intense peaks were observed at 1342 cm^−1^ and 1101 cm^−1^, indicative of C-H bending and C-O stretching vibrations [32], respectively. However, no significant changes in the PHB distinctive bands were noted in the plasticized films, suggesting that 20 wt.% plasticizers did not alter the molecular structure of PHB.

**Figure 2:**
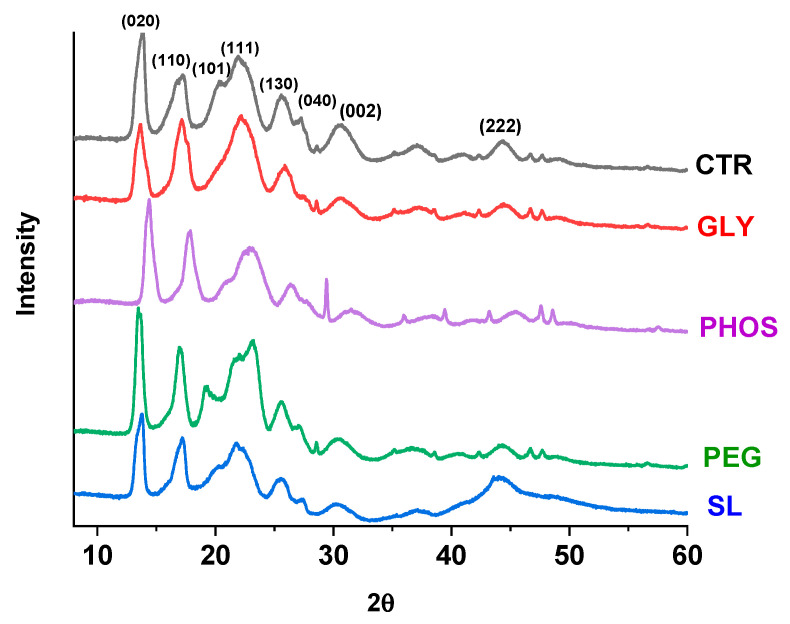
XRD of PHB films plasticized with 20 wt.% content.

X-ray diffraction (XRD) analysis was employed to investigate the effect of plasticizer-based additives on the PHB crystalline structure. Figure 2 displays the diffractograms of the pristine PHB and samples treated with various additives. The XRD pattern for pristine PHB exhibited two prominent peaks at 13.7° and 17.2°, corresponding to the (020) and (110) planes, respectively [33]. Additionally, less intense peaks were observed at 20.3°, 21.9°, 25.6°, 27.3°, 30.6°, and 44.3°, aligning with the (101), (111), (130), (040), (002), and (222) planes, respectively. The diffractograms of samples processed with different plasticizers closely resembled that of pristine PHB, indicating the retention of the same reflection peaks. Although the incorporation of the studied plasticizers affected the crystalline structure of the PHB, the diffractograms displayed peaks with reduced intensity, particularly in the PHB samples containing SL, GLY, and PHOS.

The PHB crystallinity index (CI) declined from 38% to 35%, 34%, and 33% for the PHB samples with soy lecithin, glycerol, and potassium phosphate, respectively. In contrast, the PHB-PEG samples exhibited a CI of 39.2%. Moreover, the reduction in peak intensity suggests that these plasticizers diminished the degree of crystallinity within the PHB matrix.

Quispe et al. [20] reported different findings in their studies on glycerol-plasticized PHB, indicating that incorporating GLY did not influence the crystalline structure of PHB. This discrepancy is attributable to the variations in the plasticizer concentration within the PHB matrix used in our experiments. Notably, PHB-PEG displayed identical diffraction peaks at 2θ = 19.3°, matching those of pristine PEG, affirming a consistent crystalline structure between PEG and PHB [34]. This outcome aligns with the findings reported by Panaitescu et al. [35], who stated that PEG did not hinder PHB crystallization. Research studies have not unveiled the influence of soy lecithin (SL) and potassium phosphate (PHOS) on PHB crystallinity yet.

### 3.2. Morphology Characterization

**Figure 3:**
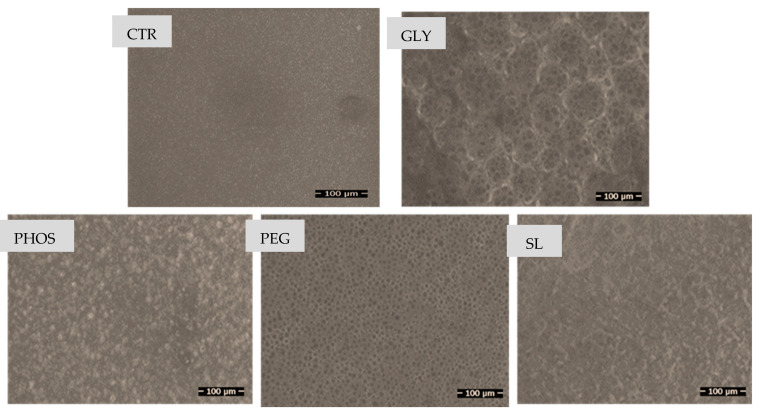
SEM micrographs of PHB films plasticized with different plasticizers: CTR (control), GLY, PHOS, PEG, and SL.

Figure 3 displays the scanning electron microscopy (SEM) images of PHB films incorporating the various plasticizers studied.

Pristine PHB exhibited a remarkably smooth and homogeneous surface morphology devoid of any discernible pores, aligning with the findings of the literature on PHBs [20,36]. In contrast, Mohamed et al. observed an irregular fracture surface in neat PHB due to its crystalline structure [37], and SEM images of the blends revealed a diverse range of flaws, predominantly in the form of holes of various shapes and sizes, following plasticizer incorporation. The plasticizers increased the roughness of PHB films, indicating a distinct rearrangement of PHB molecules provoked by specific interactions with these less polar plasticizers, yielding distortions in the PHB crystal forms.

SEM images of the PHB-GLY blend film pictured numerous holes grouped in clusters, a phenomenon previously attributed by Quispe et al. [20] to the evaporation of additive drops during electron beam sample observation. The films containing PEG exhibited non-interconnected ordered micropores, consistent with prior findings [20,38]. In this context, Ribeiro Lopes et al. [38] suggested that the absence of interconnected pores may be associated with microvoids resulting from solvent tension during the casting and drying stages of the film preparation process. However, PHOS did not significantly influence the PHB microstructure, displaying no consequential changes compared to the micrograph of pure PHB. Contrarily, SEM images of the PHB-SL blend film revealed a spongy structure, yet the effect of these two plasticizers on the PHB microstructure remains unexplored in the current literature.

### 3.3. Thermal Properties

**Figure 4:**
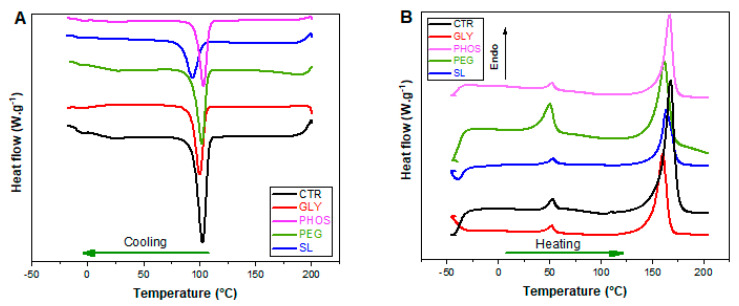
DSC curves of PHB films made with different plasticizers: (**A**) cooling run; (**B**) second heating run.

The investigated films underwent comprehensive thermal property analyses utilizing differential scanning calorimetry (DSC) and thermogravimetric analysis (TGA). Figure 4 shows the DSC curves illustrating the cooling and the second heating runs for both pristine PHB and the PHB plasticized with GLY, PHOS, PEG, and SL. Table 1 summarizes the key parameters obtained from the cooling/heating runs after the erasure of the thermal history.

The crystallization temperature (T_c_) of the PHB blends decreased slightly when plasticizers were added, except for PHB-PHOS. The most significant drop occurred upon the addition of SL, leading to a 9 °C decrease in T_c_ for the pure PHB films. This reduction is attributable to a strong plasticization effect, where the interaction hinders the PHB crystallization process [24].

The DSC heating thermogram of pure PHB revealed two distinctive peaks in the melting region: a minor endotherm around 52.6 °C and a prominent melting peak at 168.2 °C (Figure 4B). The origin of the first small peak can be attributed to various additives, low-molecular-weight polyhydroxybutyrate, or medium-chain PHAs, exhibiting low melting temperatures (T_m_). Alternatively, it could have arisen from enthalpy recovery due to the structural relaxation of the amorphous phase during the storage period before testing [35]. The introduction of plasticizers did not induce substantial alterations in the small endotherm, except for the PHB-PEG blend, where an upsurge in peak intensity was observed. This aligns with the findings of Panaitescu et al. [35], emphasizing the significant influence of PEG on the small endotherm, possibly due to the commercial-grade PHB containing this additive. The second peak was attributed to the most ordered PHB crystals. The addition of plasticizers led to a slight decrease in the T_m_ of the samples, thereby expanding the processing temperature window and enhancing PHB processability [24]. Similar behavior was observed in other PHB systems.

Other PHB systems exhibited similar behavior. For example, Frone et al. [39] noted an apparent depression of T_m_ values upon adding Tributyl 2-acetyl citrate (TAC), attributable to TAC’s ability to favor the segmental motion of the lower-molecular-weight PHB chains. The most significant drop in T_m_ occurred with GLY, where T_m_ fell to 160.5 °C. This reduction is potentially attributable to the effect of glycerol in weakening the intermolecular forces between adjacent PHB chains, leading to a change in free volume and a consequential decline in melting temperature [15]. Fusion enthalpy (∆H_2_) also dropped from 83.0 to 51.7 J·g^−1^, except for the PHB-PHOS sample. A decrease in the degree of crystallinity (X_DSC_) in the PHB blends indicated a direct interaction between the plasticizers and the PHB matrix. This reduction was particularly pronounced in the PHB-SL sample, showing an X_DSC_ up to 50% lower than pristine PHB, potentially attributed to a dilution effect [24]. According to de Sousa Junior et al. [24], the disparity between the crystallinities observed in DSC and XRD is ascribable to methodological differences: XRD emphasizes surface crystallinity, while DSC reflects bulk behavior.

**Figure 5:**
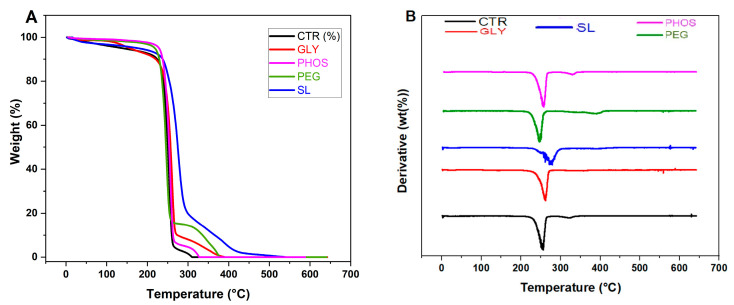
TGA (**A**) and DTG (**B**) curves of PHB films plasticized with 20 wt.% content.

The thermogravimetric analyses (TGA) examined the influence of the relevant plasticizers on the thermal stability of PHB films. Figure 5 illustrates the weight loss curves (Figure 5A) and their corresponding derivative curves (Figure 5B) for pristine and plasticized PHB films. Table 2 provides the thermal degradation parameters extracted from these curves. The pristine PHB degradation (CTR) occurred in two distinct steps, with the first and second decomposition temperatures at 256 °C and 322 °C, respectively.

The existing literature attributes the initial weight loss event to the PHB random chain scission through intramolecular cis-elimination [24], while the second weight loss is linked to the degradation of additives, excluding the plasticizer or crosslinking reactions involving PHB and additives [35]. For pristine, PHB temperatures corresponding to 5% and 10% weight loss were 138 °C and 224 °C, respectively. These values increased uniformly in the presence of plasticizers. Notably, samples treated with SL, GLY, and PHOS exhibited a significant upsurge in T_d1_, particularly evident in the PHB-SL blend film, suggesting a delay in the scission of PHB chains and a marked amelioration in thermal stability. Conversely, Quispe et al. [20] reported a pro-degradative impact for PHB-GLY blend films, assignable to glycerol readiness for transesterification reactions and the consequential reduction in decomposition temperatures due to chain reduction. Regarding the second weight loss event, the intensity of the peaks corresponded to the weight fraction of each component, with the highest decomposition temperature reaching 385 °C for 20% SL. When the PEG was used as a plasticizer, a third degradation temperature was recorded at 391 °C. This aligns with the plasticizer degradation [24]. This result demonstrates that the PHB-PEG film exhibits the greatest resistance to thermal degradation, which is an important property for materials in a variety of applications. However, Parra et al. [15] suggested that employing 20% PEG could promote the diffusion of the plasticizer to the surface through heating, reducing the thermal stability of the films. Incorporating the plasticizers significantly boosted the thermal stability of PHB films by increasing T_d2_. Comparable findings were reported by de Sousa Junior et al. [24] in their study of PHB incorporating two plasticizers derived from different reactions. They emphasized that adding plasticizers did not induce PHB degradation due to the enhanced thermal stability of the plasticizer compared to that of PHB [40].

### 3.4. Surface Wettability

**Figure 6:**
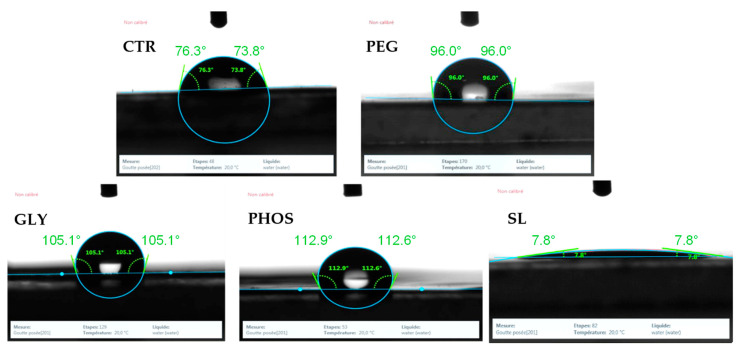
Contact angle of the produced films: CTR (control), PEG, GLY, PHOS, and SL.

The changes in PHB surface physicochemical properties due to plasticizer incorporation were analyzed using the drop shape analysis (Figure 6). Water contact angle measures a solid’s surface wettability. A low water contact angle (θ < 90°) indicates good wettability, meaning the surface is hydrophilic, while a high contact angle (θ > 90°) indicates poor wettability, inferring a hydrophobic surface [41]. For PHB, the surface roughness, crystallinity, and the presence of additives can influence the water contact angle.

The contact angle measurement of the pristine PHB film initially revealed a hydrophilic surface, as shown in Figure 6. This observation aligns well with the findings of Arrieta et al. [35], who also confirmed the hydrophilic characteristics of pristine PHB surfaces. The introduction of plasticizers into the PHB matrix raised the water contact angle from 76.9° to 94.8°, 103.9°, and 108.9° for PEG, GLY, and PHOS, respectively. Accordingly, the films became more hydrophobic after adding the plasticizer, implying that these plasticized surfaces do not readily absorb water. This change is particularly consequential for the food packaging industry, where products must be protected from moisture during transport and storage [42]. Protection against humidity is essential for items such as snacks and bakery products. In contrast, SL significantly decreased the water contact angle (7.8°), suggesting a substantial rise in the hydrophilic nature of the PHB matrix upon SL incorporation, which allowed water to spread more readily. This trend is consistent with the findings obtained from other plasticized PHB systems, where Arrieta et al. [35] suggested that the diverse effects of plasticizers on PHB contact angles are attributable to the variations in their water solubility. In addition, increased polymer chain mobility might influence the water diffusion process [43]. Hence, SL exhibited distinctive interaction within the PHB matrix, displaying a strong affinity for water, likely due to its amphiphilic properties. Hydrophilicity significantly impacts packaging characteristics in the food industry. For instance, in applications requiring short-term water absorption, such as vegetable and fruit packaging, the hydrophilic surfaces can absorb moisture, prolonging the freshness of the food without diffusing harmful chemicals. Furthermore, hydrophilic surfaces can also promote adhesion with ink and labels.

### 3.5. Optical Properties

Transparency is a critical parameter assessing films for use in food packaging. The transparency metrics for films incorporating various plasticizers are given in Table 3. Notably, the pristine PHB film demonstrated the highest transparency level, quantified at approximately 17.62, aligning with the findings of Tănase et al. [28]. Table 3 points out that the addition of plasticizer generated a discernible reduction in transparency, leading to a definite increase in opacity, which was particularly significant for PEG. This result indicates that PEG incorporation substantially attenuated light transmission through the PHB matrix, as noticed in analogous PHB systems integrating cellulose fibers where consequential alterations in the visible spectrum present reduced transparency [28].

The favorable increment in opacity renders these films eminently suited for safeguarding light-sensitive food constituents, encompassing vitamins, lipids, flavors, and pigments [32]. Compared to their plasticized counterparts, the introduction of PHOS within the PHB matrix yielded the most transparent film. This enhanced transparency is ascribable to the minimal effect of PHOS on the intermolecular forces, resulting in a slight reduction in the compactness of the matrix. Similarly, the superior dispersion of PHOS within the PHB matrix relative to other plasticizers potentially contributed to this augmented transparency.

### 3.6. Mechanical Properties

**Figure 7:**
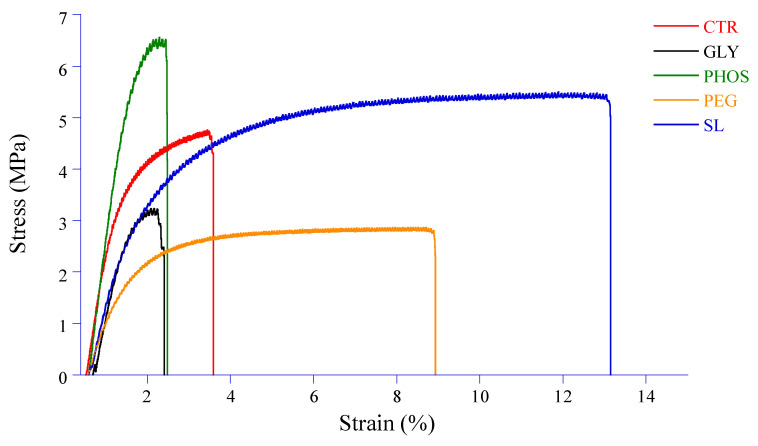
Effect of different plasticizers on the stress vs. strain curves.

**Figure 8:**
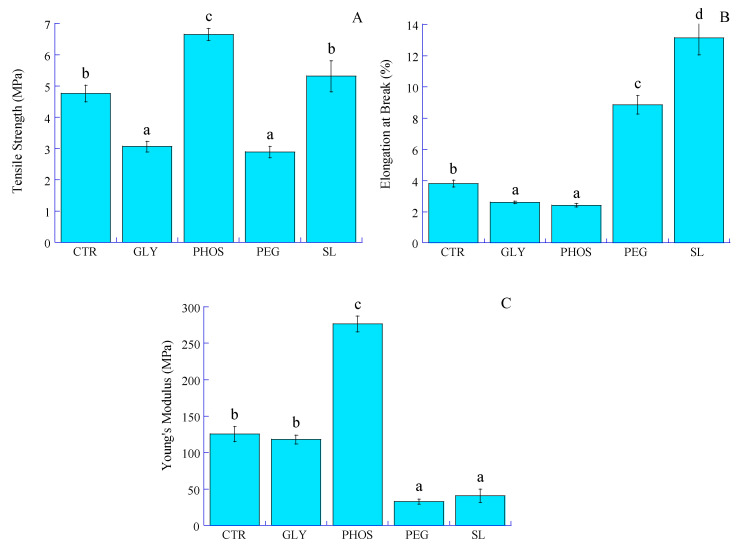
Effect of different plasticizers on the (**A**) tensile strength, (**B**) elongation at break, and (**C**) Young’s modulus of the PHB films. Bars with different letters in the figure are significantly different (*p* < 0.05).

Despite its high crystallinity, the limited mechanical strength of PHB has impeded its potential use in food packaging applications. Plasticization becomes imperative to improve the structural integrity of the film and its resistance to external forces. Key mechanical properties such as tensile strength (TS), elongation at break (EAB), and Young’s modulus (E) play a crucial role in helping packaging materials withstand stress during storage, processing, and handling. Figure 7 illustrates the stress vs. strain curves, and Figure 8 shows the impact of plasticizers on the TS, EAB, and E of PHB films.

While tensile strength is the maximum stress a film can withstand when stretched before breaking, Young’s (or elastic) modulus measures the material stiffness given by the slope of the linear domain (often observed at low deformations) of a stress–strain curve (Figure 7).

The pristine PHB film exhibits a low TS combined with a high E, indicating that the PHB film is both brittle and stiff due to its highly crystalline nature. This result aligns with that found by Sousa Junior et al. [24], which might restrict its use where durability and tear resistance are essential. The elongation at break of the pristine PHB film, which is a measure of its ability to stretch before breaking, is low (Figure 8). The tensile strength values of PHB films significantly decreased from 4.8 to 2.9 and 3.1 MPa when incorporating 20% PEG and GLY into the PHB film, respectively. This decrease resulted from the sound compatibility of PEG and GLY with PHB, allowing the plasticizer to interact within the PHB matrix through hydrogen bonding. Similar observations were reported by Parra et al. [15], ascribing to PEG the ability to reduce constraints during testing, potentially inhibiting chain motion or weakening the secondary intermolecular bonds between PHB chains. The addition of plasticizers considerably decreased Young’s modulus, notably for PEG, with a 74% decrease compared to pristine PHB. However, adding 20% PHOS resulted in a significant decline in EAB from 3.8 to 2.4%, accompanied by an increase in TS and E values. As for GLY, despite a decrease in TS, the EAB value significantly dropped from 3.8 to 2.6%, while E seemed slightly influenced. Comparable results were reported by Quispe et al. [20], who attributed PHB thermal degradation induced by GLY to chain shortening and advised caution in its use due to its adverse effects on mechanical properties. On the other hand, soy lecithin seemed to have a neutral effect on the tensile strength of PHB-based films. Moreover, the increase in elongation at break from 3.8 to 13% and a fall in Young’s modulus from 125.3 to 40.9 MPa for PHB-SL could be assigned to reduced inter-macromolecular interactions and increased PHB chain mobility. This observation suggests a more cohesive three-dimensional structure facilitated by soy lecithin amphiphilic molecules acting as stretch bridges and allowing for deformation without rupture. These results are consistent with the decrease in crystallinity indexes (CI and X_DSC_) and the increase in degradation temperatures (T_d1_ and T_d2_) observed upon adding SL to PHB. However, the influence of soy lecithin and potassium phosphate on PHB mechanical properties remains unreported in the current literature.

## 4. Conclusions

This study investigated the characteristics of four plasticized films: glycerol, polyethylene glycol, potassium phosphate, and soy lecithin. This investigation revealed no chemical interaction between the plasticizers and PHB, and the molecular structure of PHB remained unchanged upon the incorporation of the plasticizers. Upon including SL, GLY, and PHOS particles, the XRD characterization revealed a reduction in crystallinity. In the presence of different plasticizers, SEM images of PHB–plasticizer blends showed a wide range of defects, primarily holes in distinct sizes and forms. While DSC revealed a decrease in T_m_, particularly in the case of SL, TGA showed that all plasticizers considerably enhanced the PHB thermal stability. Apart from the film containing 20% PEG, all plasticizers somewhat reduced PHB transparency. Soy lecithin was the most promising plasticizer to incorporate in the PHB film because of its hydrophilic character. Soy lecithin is ultimately the most promising of the four plasticizers studied for future usage in film production due to its high heat stability and a comparatively high elongation of 13%. Stretchable hydrophilic PHB-SL films can be wrapped around perishable foods like vegetables and fruits and protect them while maintaining humidity. This prevents excessive water vapor condensation, which could be the origin of food deterioration.

## Figures and Tables

**Table 1:** DSC results of PHB films with various plasticizers.

Plasticizer	T_c_ (°C)	∆H_c_ (J·g^−1^)	T_m1_ (°C)	∆H_m1_ (J·g^−1^)	T_m2_ (°C)	∆H_m2_ (J·g^−1^)	X_DSC_ (%)
Control	102.8	−71.4	58.6	4.0	168.2	83.05	56.9
GLY	100.2	−56.9	52.1	3.8	160. 5	69.8	38.2
PHOS	103.6	−72.3	53.2	5.3	166.8	86.1	47.2
PEG	102.4	−62.5	52.2	24.0	162.1	80.2	43.9
SL	93.8	−44.5	50.0	3.8	164. 2	51.7	28.3

T_c_: crystallization peak temperature. ∆H_c_: enthalpy of crystallization. T_m_: melting peak temperature. ∆H_m_: enthalpy of melting. X_DSC_: degree of crystallinity. Subscripts: 1 for cooling run, 2 for second heating run.

**Table 2:** TGA results of PHB films.

Plasticizer	T_5%_ (°C)	T_10%_ (°C)	T_d1_ (°C)	T_d2_ (°C)	T_d3_ (°C)
Control	138	224	256	322	−
GLY	156	220	262	357	−
PHOS	229	237	258	330	−
PEG	218	229	247	343	391
SL	176	238	279	385	−

T_5%_: 5% weight loss temperatures. T_10%_: 10% weight loss temperatures. T_d1_, T_d2_, and T_d3_: first, second, and third degradation temperatures, respectively.

**Table 3:** Transparency of PHB films.

Plasticizer	Control	GLY	PHOS	PEG	SL
Transparency	17.62 ± 0.17 ^e^	14.23 ± 0.11 ^b^	16.99 ± 0.16 ^d^	12.80 ± 0.13 ^a^	16.25 ± 0.03 ^c^

Columns with different superscript letters are significantly different (*p* < 0.05).

## Data Availability

The original contributions presented in this study are included in the article. Further inquiries can be directed to the corresponding authors.
